# Treatment Outcomes of Catheter Ablation Versus Medical Therapy in Patients With Atrial Fibrillation: A Systematic Review

**DOI:** 10.7759/cureus.60340

**Published:** 2024-05-15

**Authors:** Moeed Ali Karim, Wei-Hsun Huang

**Affiliations:** 1 Medicine, University of Sydney, Sydney, AUS; 2 General Medicine, Capital Medical University, Beijing, CHN

**Keywords:** systematic review, rhythm control, medical therapy, catheter ablation, atrial fibrillation

## Abstract

Atrial fibrillation (AF) management has witnessed a paradigm shift, with an increasing emphasis on rhythm control strategies. This systematic review aims to comprehensively assess and compare the efficacy and safety of catheter ablation versus medical therapy in the treatment of AF. A systematic search was conducted across major electronic databases, including PubMed, Embase, and the Cochrane Library, from inception to the present. Randomized controlled trials (RCTs) and observational studies comparing catheter ablation with medical therapy for AF were included. The primary outcomes included rhythm control success, recurrence rates, and adverse events. Secondary outcomes encompassed quality of life, hospitalization rates, and mortality. A total of six studies met the inclusion criteria, comprising 2,859 participants. Catheter ablation significantly improved rhythm control success compared to medical therapy. Subgroup analyses demonstrated variations in outcomes based on patient characteristics, procedural techniques, and follow-up durations. Recurrence rates favored ablation; however, ablation was associated with a higher incidence of minor complications and major adverse events. Catheter ablation demonstrates superior efficacy in achieving and maintaining rhythm control compared to medical therapy in the management of AF. Despite the increased risk of procedural complications, the overall safety profile remains acceptable. This systematic review provides valuable insights for clinicians and informs shared decision-making between patients and healthcare providers when choosing between catheter ablation and medical therapy for AF treatment.

## Introduction and background

Atrial fibrillation (AF) stands as the most prevalent sustained cardiac arrhythmia globally, affecting approximately 2-3% of the general population worldwide and posing a significant burden on healthcare systems [1]. Its prevalence increases with age, with estimates suggesting that about 9% of individuals aged 65 years or older have AF [2]. Characterized by irregular and rapid atrial contractions, AF disrupts the normal rhythm of the heart, leading to impaired cardiac function and an increased risk of thromboembolic events [3]. As a result, AF contributes substantially to healthcare utilization and costs. Hospitalizations related to AF, particularly for stroke and other cardiovascular complications, impose a significant economic burden on healthcare systems globally [4]. Therefore, as the prevalence of AF continues to rise with the aging population, the need for optimal management strategies has become apparent.

The complexity of AF arises from its multifactorial etiology, often intertwined with structural heart disease, hypertension, valvular disorders, and other contributing factors [5]. Thus, the treatment landscape for AF is evolving rapidly, reflecting the complex nature of this arrhythmia. Historically, treatment approaches have predominantly centered on rate and rhythm control through pharmacological interventions [6]. However, recent years have seen a paradigm shift toward rhythm control strategies, with catheter ablation emerging as a promising option.

Catheter ablation involves targeted destruction of aberrant electrical pathways within the heart, particularly focusing on the pulmonary veins, with the aim of restoring and maintaining normal sinus rhythm [6]. This nonpharmacological intervention offers a potential cure for AF and has evolved significantly, with refinements in techniques and technologies demonstrating efficacy in select patient populations [7]. Importantly, catheter ablation addresses the limitations of medical therapy, which often entails a balance between achieving rhythm control and managing the potential side effects of antiarrhythmic drugs (AADs) [8].

Nevertheless, medical therapy remains a cornerstone in AF management, encompassing a range of pharmacological agents targeting heart rate, rhythm, and thromboembolic event prevention. AADs, such as amiodarone and flecainide, are integral to rhythm control, while anticoagulants, including warfarin and direct oral anticoagulants, are essential for stroke prevention [9]. The integration of catheter ablation and medical therapy highlights the importance of a multidisciplinary approach tailored to individual patient characteristics and preferences, paving the way for improved outcomes in AF management.

Despite the growing body of evidence supporting the efficacy of catheter ablation compared to medical therapy in managing AF, there are notable gaps in the current literature that warrant attention. There is a need for more robust comparative effectiveness research incorporating diverse patient populations, including those with comorbidities such as heart failure (HF), advanced age, and prior stroke. Furthermore, while certain randomized controlled trials (RCTs) have demonstrated the superiority of catheter ablation over medical therapy, there remains variability in procedural success rates and outcomes across different centers and operators, highlighting the importance of standardizing procedural techniques and optimizing patient selection criteria. Addressing these gaps in the literature is crucial for informing clinical decision-making and optimizing treatment strategies for patients with AF. This systematic review aims to comprehensively assess and compare the efficacy and safety of catheter ablation versus medical therapy in the treatment of AF.

## Review

Methods

This systematic review adhered to the Preferred Reporting Items for Systematic Reviews and Meta-Analyses (PRISMA) guidelines and followed the Grading of Recommendations, Assessment, Development, and Evaluations (GRADE) criteria for assessment [10].

Search Strategy

A comprehensive search strategy was developed and implemented for the literature databases PubMed, Scopus, and Web of Science by an independent author between February 26 and February 29, 2024. The search terms “AF”, “catheter ablation”, “medical therapy”, “treatment outcomes”, procedural success”, “rhythm control”, “rate control”, “recurrence”, “complications”, “mortality”, “quality of life”, “cost-effectiveness”, “randomised controlled trial”, “patient satisfaction”, and “long term” were used to frame the initial searches.

Inclusion and Exclusion Criteria

Literature was then evaluated for relevance against the inclusion and exclusion criteria. Studies were included if they (a) followed an RCT design; (b) comprised adult patients (18 years of age or older) diagnosed with AF; (c) conducted a comparison of catheter ablation with medical therapy; and (d) reported on treatment outcomes, including but not limited to the maintenance of sinus rhythm, reduction in AF recurrence, quality of life improvement, cardiovascular events, and mortality rates. Literature predating 2014 was omitted to maintain relevance to contemporary practices, as was literature not in English. Furthermore, reference lists from identified literature and previous review articles were scrutinized to gather further pertinent material.

Study Selection and Data Extraction

An independent reviewer conducted the initial screening of titles and abstracts to identify potentially eligible studies. Full-text articles of potentially relevant studies were obtained and reviewed against the inclusion and exclusion criteria. All the identified and relevant literature was then imported into EndNote X9 for ease during the screening process. A standardized data extraction form was developed and piloted. An independent reviewer extracted data from selected studies and included study characteristics (author, publication year, and study design), participant demographics, details of interventions (catheter ablation and medical therapy protocols), and treatment outcomes. Outcome data included measures of rhythm control, symptom improvement, quality of life, and reported adverse events.

Data Synthesis

Quantitative data, if homogenous, will be pooled for meta-analysis using statistical software. If significant heterogeneity exists, a narrative synthesis will be conducted. Subgroup analyses may be performed based on the study design, patient characteristics, or other relevant factors.

Risk of Bias and Quality Assessment

The quality of the literature included was assessed according to GRADE criteria [11], which assess methodological quality, directness of evidence, heterogeneity, precision of effect estimates, and risk of publication bias. This evaluation resulted in assigning a score indicating a high, moderate, or low level of evidence and a recommendation for use.

Results

Identification of the Literature

**Figure 1:**
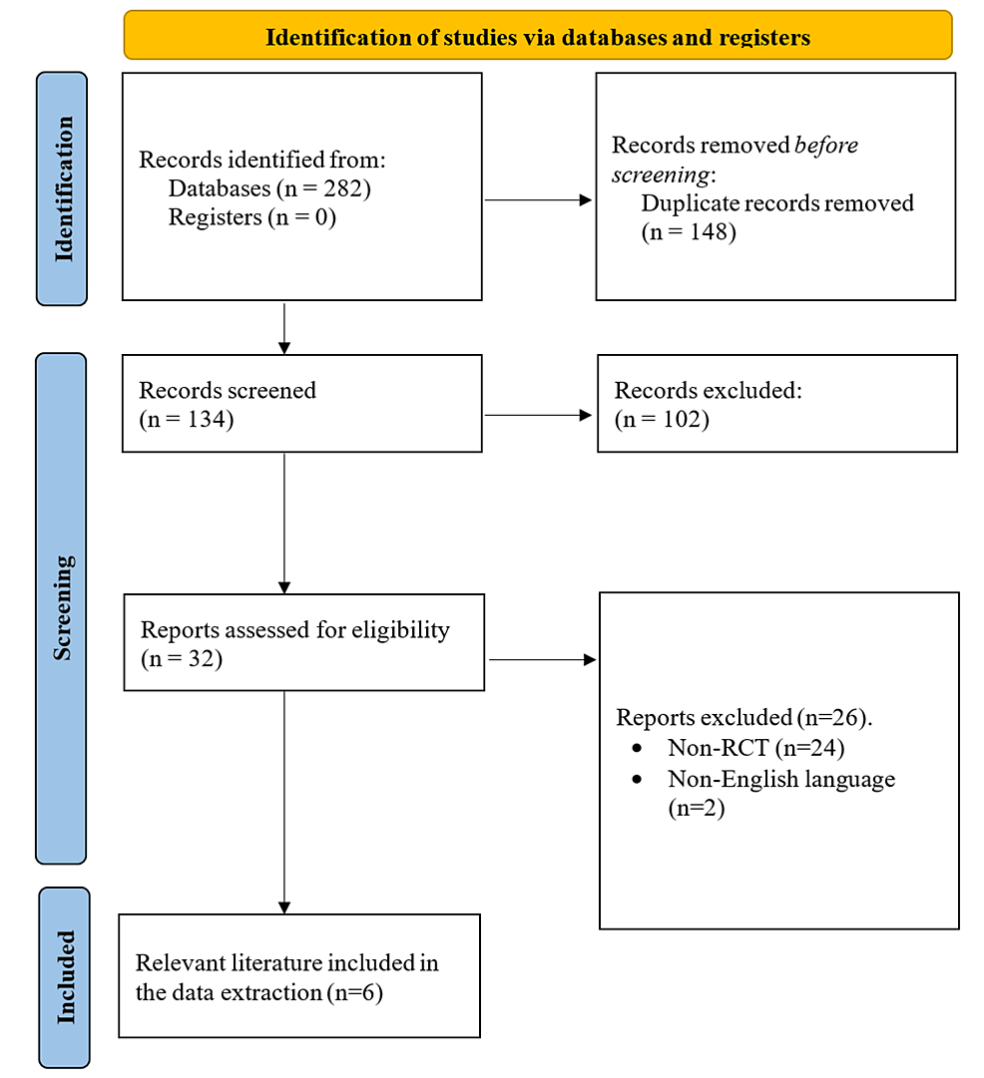
PRISMA flow diagram depicting the literature search and study selection process Reports were excluded as they did not meet the inclusion or exclusion criteria. PRISMA, Preferred Reporting Items for Systematic Reviews and Meta-Analyses; RCT, randomized controlled trial

After implementing the search strategy outlined, a total of 282 records were initially identified as relevant to the research question. Subsequently, duplicate records were removed using EndNote X9, resulting in 134 unique records. These records underwent screening based on their titles and abstracts against predefined inclusion and exclusion criteria, leading to the exclusion of 102 records. The remaining 32 records underwent full-text assessment for eligibility, during which 26 were excluded due to not being RCTs or not being published in the English language. As a result, six full-text articles were deemed relevant and included in both the qualitative and quantitative analyses of this systematic review (refer to Figure 1 for details).

All the included literature investigated the outcomes of catheter ablation compared to medical therapy for the treatment of AF. A total of 2,859 patients were included, who were divided into separate groups to receive medical therapy or catheter ablation. The methodology followed a similar pattern across the included literature.

Impact of Catheter Ablation Compared to Medical Therapy

The findings of the included RCTs highlight the general superiority of catheter ablation in managing AF compared to traditional medical therapy, although not all findings demonstrate significant differences.

Early rhythm control catheter ablation (ER-CA) resulted in improved symptom scores, functional capacity, and left ventricular ejection fraction (LVEF) compared to rate control (RC) alone. ER-CA showed similar long-term outcomes to delayed strategy catheter ablation (DS-CA), but treatment received analysis suggested improved outcomes with CA versus RC [12]. Additionally, catheter ablation led to clinically significant improvements in quality of life compared to medical therapy, as evidenced by higher AF Effect on QualiTy-of-life (AFEQT) and lower Mayo AF-specific Symptom Inventory (MAFSI) scores at 12 months in patients with symptomatic AF [12]. Similarly, catheter ablation significantly increased the proportion of patients free from any episode of AF or atrial flutter lasting >24 hours compared to AAD therapy. Cardioversion was also less frequent in the catheter ablation group [13].

Among secondary endpoints, catheter ablation showed a trend toward lower all-cause mortality compared to drug therapy (5.2% vs. 6.1%). However, it significantly reduced the incidence of death or cardiovascular hospitalization (51.7% vs. 58.1%) and AF recurrence (49.9% vs. 69.5%). The incidence of death, disabling stroke, serious bleeding, or cardiac arrest did not significantly differ between the catheter ablation and medical therapy groups [14]. Radiofrequency ablation also significantly reduced the rate of persistent AF/AT compared to AAD therapy at three years. Patients ≥65 years had a higher risk of progression to persistent AF/AT. RF ablation was superior to AAD therapy in delaying the progression from paroxysmal to persistent AF [15].

However, the Atrial Fibrillation Management in Congestive Heart Failure With Ablation (AMICA) trial revealed no benefit of catheter ablation over medical therapy in patients with AF and advanced HF at one year. Both groups showed similar improvements in LVEF, with comparable rates of sinus rhythm and AF burden [13].

Quality Assessment

Packer et al. (2019) had limitations due to patient withdrawals and crossovers between treatment groups, potentially affecting estimates of treatment effects and precision. However, ablation techniques remained largely consistent throughout the trial [14]. This warranted a moderate quality scoring due to limitations in the study design and potential biases. Kuck et al. (2019) attained a low quality of evidence due to the premature termination of the study and missing data for the primary endpoint, which reduced the ability to adequately address the study hypothesis. The observed increase in LVEF in the ablation arm was also lower than projected [15].

Kuck et al. (2021) achieved a moderate quality of evidence due to limitations in generalizability and potential confounding factors. The study used only RF catheters, limiting the generalizability of the results to other ablation technologies. Additionally, the initiation of new AAD regimens during the study may have introduced confounding factors [16]. Mark et al. (2019) presented moderate quality despite limitations in masking and missing data due to robust statistical analysis. Limitations included a lack of masking in treatment groups and potential biases due to missing follow-up data. However, the study employed a robust statistical approach to ensure the reliability of the results over time [17].

Zakeri et al. (2023) presented a low quality of evidence due to the small cohort size and wide CIs. The small cohort size resulted in wide CIs, limiting the precision of the estimated treatment effects. However, the study provided unique long-term data on HFrEF patients [18]. Mont et al. (2014) terminated the study prematurely, potentially impacting statistical power. However, blinded evaluation of endpoints and pragmatic monitoring approaches were employed to mitigate biases, warranting a moderate quality of evidence score [19]. A summary of the GRADE scoring and quality assessment is presented in Table 1.

**Table 1:** Data extraction of identified literature AAD, antiarrhythmic drug; ADT, antiarrhythmic drug therapy; AF, atrial fibrillation; AFEQT, atrial fibrillation effect on quality of life summary score; aHR, adjusted hazard ratio; AT, atrial tachycardia; BMT, bone marrow transplantation; CA, catheter ablation; DS-CA, direct sense catheter ablation; ER-CA, emergency recurrence catheter ablation; HR, hazard ratio; LVEF, left ventricular ejection fraction; MAFSI, Mayo AF-specific Symptom Inventory; NT-proBNP, N-terminal pro b-type natriuretic peptide; RC, rate control; RCT, randomized controlled trial; RF, radiofrequency

Study	Study design	Intervention	Follow-up duration	Primary outcome(s)	Secondary outcome(s)	Key findings	Quality assessment
Packer et al. (2019) [14]	RCT	Catheter ablation	48.5 months	Composite of death, disabling stroke, serious bleeding, and cardiac arrest	All-cause mortality, total mortality, cardiovascular hospitalization, and AF recurrence	The main outcome was observed in 8.0% (n = 89) of patients receiving ablation compared to 9.2% (n = 101) of patients receiving drug therapy (HR: 0.86 (95% CI, 0.65-1.15); P = 0.30)	Moderate
Kuck et al. (2019) [15]	RCT	Catheter ablation	358 days	Absolute increase in LVEF from baseline at one year	Six-minute walk test, quality of life, and NT-proBNP	By the one-year mark, LVEF had risen by 8.8% (95% CI, 5.8-11.9%) in patients who underwent ablation and by 7.3% (4.3-10.3%) in those receiving BMT (P = 0.36)	Low
Kuck et al. (2021) [16]	RCT	Catheter ablation	3 years	Rate of persistent AF/atrial tachycardia at three years	Rate of persistent AF/atrial tachycardia at one and two years, time to recurrent AF/AT, number of repeat ablations, and new AAD	At the three-year mark, the incidence of persistent AF/AT was markedly reduced with RF ablation (2.4% (95% CI, 0.6-9.4%)) compared to AAD therapy (17.5% (95% CI, 10.7-27.9%); one-sided P = 0.0009)	Moderate
Mark et al. (2019) [17]	RCT	Catheter ablation	48.5 months	AFEQT score and MAFSI frequency score	N/A	After 12 months, the catheter ablation group showed a more positive mean AFEQT summary score compared to the drug therapy group (86.4 points vs. 80.9 points) (adjusted difference, 5.3 points (95% CI, 3.7-6.9); P < 0.001)	Moderate
Zakeri et al. (2023) [18]	RCT	Catheter ablation	7.8 years	All-cause mortality and hospitalization due to cardiovascular causes	N/A	When compared to DS-CA, adopting an ER-CA strategy showed a comparable risk of all-cause mortality (aHR 0.89, 95% CI 0.44-1.77, P = 0.731) and combined all-cause mortality or cardiovascular hospitalization (aHR 0.80, 95% CI 0.43-1.47, p = 0.467). However, analyses based on treatment received suggested a correlation between CA and enhanced outcomes compared to RC (all-cause mortality: aHR 0.43, 95% CI 0.20-0.91, P = 0.028; all-cause mortality/cardiovascular hospitalization: aHR 0.48, 95% CI 0.24-0.94, P = 0.031)	Low
Mont et al. (2014) [19]	RCT	Catheter ablation	12 months	Any episode of AF or atrial flutter lasting >24 hours that occurred after a three-month blanking period	Any atrial tachyarrhythmia lasting >30 seconds, hospitalization, and electrical cardioversion	In an intention-to-treat analysis, 69 out of 98 patients (70.4%) in the CA group and 21 out of 48 patients (43.7%) in the ADT group were free of the primary endpoint (P = 0.002), indicating an absolute risk difference of 26.6% (95% CI 10.0-43.3) favoring CA. Additionally, the proportion of patients free of any recurrence (>30 seconds) was higher in the CA group compared to the ADT group (60.2% vs. 29.2%; P < 0.001), and cardioversion was less frequent (34.7% vs. 50%, respectively; P = 0.018)	Moderate

Discussion

The impact of catheter ablation compared to medical therapy in managing AF has been a subject of significant interest and research. This systematic review aimed to comprehensively assess and compare the efficacy and safety of catheter ablation versus medical therapy in the treatment of AF. Our findings underscore the potential of catheter ablation as a superior intervention for managing AF.

The findings from various RCTs generally suggest that catheter ablation is superior to traditional medical therapy for managing AF. While not all findings demonstrate significant differences, the overall trend favors catheter ablation. This aligns with current literature emphasizing the role of catheter ablation as an effective intervention for AF management, particularly in improving patient outcomes and quality of life. Early rhythm control catheter ablation has also shown notable benefits over RC alone in terms of improved symptom scores, functional capacity, and LVEF. Additionally, catheter ablation has been associated with clinically significant enhancements in quality of life compared to medical therapy, as indicated by higher AFEQT scores and lower MAFSI scores. These findings corroborate the current literature, with Rohrer et al. reporting a beneficial effect on quality of life independent of the timepoint and strategy of catheter ablation [20]. Similarly, Walfridsson et al. observed that catheter ablation had long-lasting effects on symptoms and health-related quality of life associated with AF among 1,521 patients [21].

Catheter ablation has demonstrated superiority over medical therapy in reducing the recurrence of AF and atrial flutter, as well as lowering the frequency of cardioversions. Moreover, it has shown a trend toward lower all-cause mortality and significantly reduced the incidence of death or cardiovascular hospitalization compared to drug therapy. Asad et al. conducted a similar systematic review and meta-analysis comparing these two management approaches and their outcomes in patients with AF. The findings demonstrated a significant reduction in all-cause mortality with catheter ablation and significantly fewer cardiovascular hospitalizations and recurrences of atrial arrhythmias compared to medical therapy [22].

Despite the overall positive outcomes associated with catheter ablation, challenges exist, particularly in specific patient populations. The AMICA trial, for instance, revealed no significant benefit of catheter ablation over medical therapy in patients with AF and advanced HF at one year. This highlights the importance of considering patient characteristics and comorbidities when assessing the efficacy of catheter ablation. Current literature acknowledges the need for personalized treatment approaches and further research to address the nuances of managing AF in diverse patient populations.

## Conclusions

The findings from RCTs comparing catheter ablation to medical therapy underscore the potential of catheter ablation as a superior intervention for managing AF. While challenges exist, particularly in specific patient subsets, the overall evidence supports the efficacy of catheter ablation in improving symptoms, reducing AF recurrence, and lowering cardiovascular events compared to traditional medical therapy. This discussion contributes to the ongoing dialogue surrounding the optimal management strategies for AF, emphasizing the importance of individualized treatment approaches and further research to enhance patient outcomes in this population.
